# Functional Characterization of *CsSWEET5a*, a Cucumber Hexose Transporter That Mediates the Hexose Supply for Pollen Development and Rescues Male Fertility in Arabidopsis

**DOI:** 10.3390/ijms25021332

**Published:** 2024-01-22

**Authors:** Liping Hu, Jiaxing Tian, Feng Zhang, Shuhui Song, Bing Cheng, Guangmin Liu, Huan Liu, Xuezhi Zhao, Yaqin Wang, Hongju He

**Affiliations:** 1 Institute of Agri-Food Processing and Nutrition, Beijing Academy of Agricultural and Forestry Sciences, Beijing 100097, China; huliping@iapn.org.cn (L.H.); songshuhui@iapn.org.cn (S.S.); chengbing@iapn.org.cn (B.C.); liuguangmin@iapn.org.cn (G.L.); huanl0329@163.com (H.L.); lysozyme@foxmail.com (X.Z.); 2 Beijing Vegetable Research Center, Beijing Academy of Agriculture and Forestry Sciences, Beijing 100097, China; tianjiaxing@nercv.org (J.T.); zhangfeng@nercv.org (F.Z.)

**Keywords:** cucumber, hexose transporter, plasma membrane, pollen development, SWEET

## Abstract

Pollen cells require large amounts of sugars from the anther to support their development, which is critical for plant sexual reproduction and crop yield. Sugars Will Eventually be Exported Transporters (SWEETs) have been shown to play an important role in the apoplasmic unloading of sugars from anther tissues into symplasmically isolated developing pollen cells and thereby affect the sugar supply for pollen development. However, among the 17 *CsSWEET* genes identified in the cucumber (*Cucumis sativus* L.) genome, the *CsSWEET* gene involved in this process has not been identified. Here, a member of the *SWEET* gene family, *CsSWEET5a*, was identified and characterized. The quantitative real-time PCR and *β-glucuronidase* expression analysis revealed that *CsSWEET5a* is highly expressed in the anthers and pollen cells of male cucumber flowers from the microsporocyte stage (stage 9) to the mature pollen stage (stage 12). Its subcellular localization indicated that the CsSWEET5a protein is localized to the plasma membrane. The heterologous expression assays in yeast demonstrated that *CsSWEET5a* encodes a hexose transporter that can complement both glucose and fructose transport deficiencies. *CsSWEET5a* can significantly rescue the pollen viability and fertility of *atsweet8* mutant Arabidopsis plants. The possible role of *CsSWEET5a* in supplying hexose to developing pollen cells via the apoplast is also discussed.

## 1. Introduction

In flowering plants, pollen development is critical for generating male gametophytes, which are extremely important for plant sexual reproduction and crop yield [1,2]. Pollen development is a complex developmental process that requires a high amount of metabolic energy. Sugars play critical roles in pollen development because they are major nutrients and energy sources used to sustain growth and can also act as signaling molecules to influence pollen development [1,3]. Any interference with sugar supply or transport capability can severely impair pollen development, which often leads to male sterility, causing major crop yield loss [3,4,5,6].

Pollen development occurs within the anther wall, which consists of several somatic cell layers, namely, the epidermis, the endothecium, the middle layer, and the tapetum [7,8]. Pollen cells are completely heterotrophic; namely, their growth and development rely entirely on sugars derived from the anther, which is considered a pollen sugar reservoir [1,3,8]. Structurally, pollen cells are completely symplasmically isolated from the surrounding anther tissues [1,8]. Thus, sugars, including sucrose, glucose, and fructose, in anther tissues can be unloaded into pollen cells only through the apoplasmic pathway, and this physiological process generally involves a two-step sugar transporter-mediated mechanism: sugars must first be exported from anther tissues into anther locules via a sugar exporter transporter (the first step), which is followed by uptake from anther locules into pollen cells by a sugar importer transporter (the second step) [1,6,8,9,10]. Among the three families of sugar transporters discovered in plants, Sugars Will Eventually be Exported Transporters (SWEETs) are a novel family that can mediate not only the influx but also efflux of various mono- and disaccharides across cell membranes, whereas monosaccharide transporters (MSTs) or sucrose transporters/sucrose carriers (SUTs/SUCs) can only mediate the cellular influx of sugars [6,8,9,10,11,12,13,14]. That is, SWEETs, not MSTs or SUTs/SUCs, are the only candidates for controlling the first step, while all three can participate in the second step.

Recently, several studies have confirmed that SWEETs are the key sugar exporter transporters that mediate the export of sugars from anther tissues into anther locules. In Arabidopsis (*Arabidopsis thaliana*), *AtSWEET8* (also called *RPG1*), a plasma membrane-localized glucose transporter, is expressed in the tapetum of anthers and microsporocytes/microspores/pollen grains and is involved in exporting glucose from the tapetum to anther locules [11,15]. The *atsweet8* mutant exhibited pollen abortion and severely reduced male fertility, consistent with the role of *AtSWEET8* in providing a glucose supply for pollen development [15]. *AtSWEET13* and *AtSWEET14* are strongly expressed in the epidermis and endothecium of the anthers at stages 12 and 13, when both the middle layer and tapetum have degenerated and the endothecium becomes the innermost layer of the anther wall, and function in exporting sucrose out of the endothecium into anther locules [16,17]. The double mutant *atsweet13;14* shows a reduced fertility phenotype as a result of low pollen viability, which is induced by an insufficient sucrose supply [16,17]. Like *AtSWEET8*, tomato (*Solanum lycopersicum*) *SlSWEET5b* plays an important role in exporting glucose and fructose into anther locules to support pollen development, and silencing *SlSWEET5b* expression results in male sterility [6]. However, to our knowledge, the molecular mechanism through which sugars reach anther locules to support pollen development, which is crucial not only for plant reproduction but also for global food security [6,17,18], has not been determined for other plant species.

It has been fourteen years since SWEETs were first characterized in plants [11]. Compared with MSTs and SUTs/SUCs, which typically contain 12 α-helical transmembrane domains (TMs), SWEETs are smaller and generally consist of only 7 TMs [8,11,12,13]. These seven TMs are divided into two MtN3/saliva domains, each containing three TMs (1–3 and 5–7), which are connected via the remaining TM [11]. SWEETs from various plant species are divided into four clades based on their amino acid similarity [11]. In general, the SWEETs that belong to clades I, II, and IV prefer to transport hexoses, while clade III favors sucrose [19]. In fact, SWEETs are widely distributed in plants, as studies have shown that members of the *SWEET* gene family are present in almost every sequenced plant genome. For example, a total of 17, 21, 29, 35, and 17 genes have been identified in Arabidopsis, rice (*Oryza sativa*), tomato, potato (*Solanum tuberosum*), and cucumber (*Cucumis sativus*) [11,20,21,22]. In addition to being involved in pollen development [6,15,16,17], SWEETs are associated with many essential biological processes in plants, such as phloem loading and unloading [9,23], seed filling [24], nectar secretion [25,26], fruit development [27,28,29], pollen germination [30], response to abiotic stresses [31,32,33], and plant–pathogen interactions [32,34,35,36]. Notably, studies have shown that different members of the *SWEET* gene family in the same plant vary in physiological function. For example, Arabidopsis *AtSWEET8*, *AtSWEET9*, *AtSWEET15* (also called *SAG29*), and *AtSWEET16* are essential for pollen development [11,15], nectar secretion [25], leaf senescence [37], and responses to abiotic stress [31], respectively. Thus, it is necessary to analyze the biological functions of each member of the *SWEET* gene family from the same plant to elucidate the function of SWEETs more comprehensively and in greater depth.

Cucumber, a crucial vegetable crop cultivated worldwide, is a typical unisexual flower plant with individual male and female flowers: the male flower produces stamens/pollen, and the female flower bears pistils/ovules. Cucumber *CsHT1*, which encodes a pollen-specific plasma membrane-localized hexose transporter, has been found to function in pollen germination and pollen tube growth but not pollen development [38]. Recently, Sun et al. [5] reported that the downregulation of the sucrose transporter *CsSUT1* led to a significant decrease in sucrose, glucose, and fructose contents within male cucumber flowers, causing the formation of shriveled and aborted pollen grains and ultimately male sterility. These results suggested that *CsSUT1* is involved in mediating sucrose import from anther locules into pollen cells (the second step). However, the sugar transporter that mediates sugar export from anther tissues to anther locules (the first step) in cucumber has not been identified. Among the only sugar transporters that can mediate the efflux of sugars across cell membranes, the 17 *CsSWEETs* identified in the cucumber genome [22] were the best candidates for genes that export sugars from anther tissues into anther locules. However, out of the seventeen *CsSWEET* genes, only three have been characterized in detail; *CsSWEET2* enhances cold tolerance in plants [33]; *CsSWEET7a* is involved in hexose unloading in cucumber receptacles, nectaries, and fruits [26,27]; and *CsSWEET12c* promotes plant growth and blooming [39]. None of these three genes are related to pollen development.

In this study, we cloned *CsSWEET5a*, which belongs to clade II of the *SWEET* gene family, from male cucumber flowers. We found that *CsSWEET5a* is a hexose transporter that is predominantly expressed in the plasma membrane of anthers and pollen cells from the microsporocyte stage (stage 9) to the mature pollen stage (stage 12). Moreover, the overexpression of *CsSWEET5a* significantly rescued the pollen viability and fertility of *atsweet8* mutant Arabidopsis plants. Our results demonstrated the vital importance of *CsSWEET5a* in transporting hexose from anther tissues to developing pollen, which is essential for pollen development and fertility.

## 2. Results

### 2.1. CsSWEET5a Encodes the Clade II SWEET Protein

**Figure 1:**
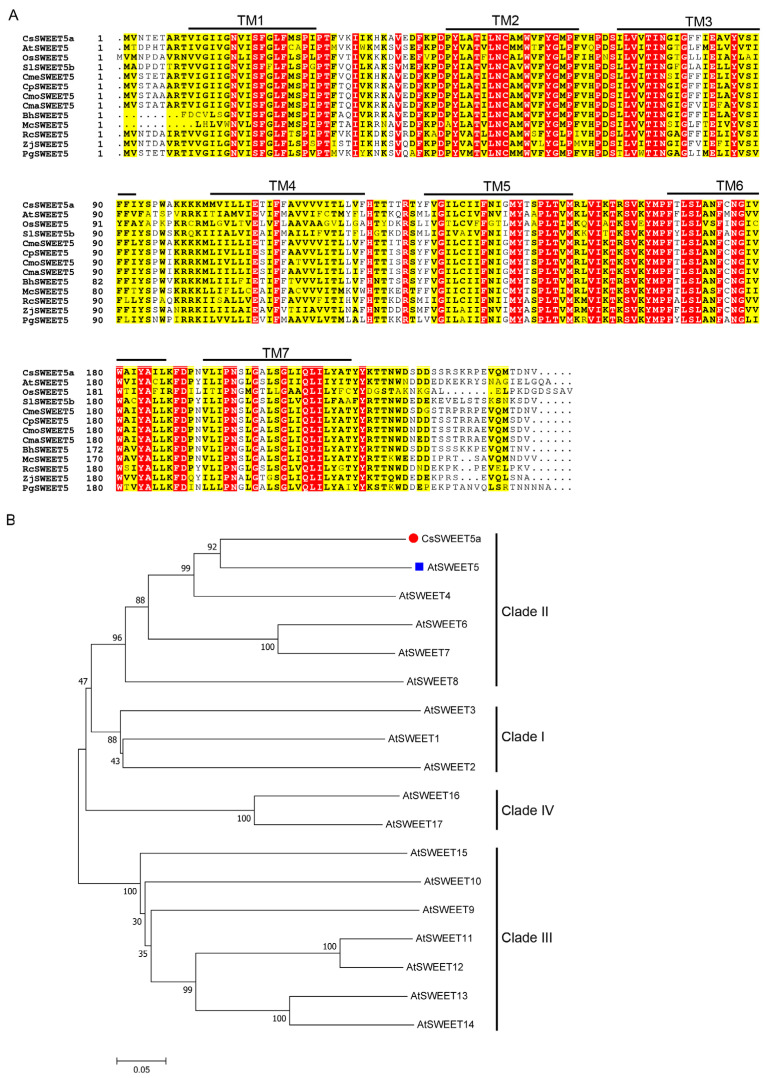
Sequence analysis of CsSWEET5a. (**A**) Multiple sequence alignment of SWEET5 proteins from *Cucumis sativus* (CsSWEET5a), *Arabidopsis thaliana* (AtSWEET5), *Oryza sativa* (OsSWEET5), *Solanum lycopersicum* (SlSWEET5b), *Cucumis melo* (CmeSWEET5), *Cucurbita pepo* (CpSWEET5), *Cucurbita moschata* (CmoSWEET5), *Cucurbita maxima* (CmaSWEET5), *Benincasa hispida* (BhSWEET2), *Momordica charantia* (McSWEET5), *Rosa chinensis* (RcSWEET5), *Ziziphus jujuba* (ZjSWEET5), and *Punica granatum* (*PgSWEET5*). The seven transmembrane domains (TMs) are outlined. The identical amino acids are denoted by white characters on a red background, and the conserved amino acids are indicated by a yellow background. (**B**) Phylogenetic analysis of SWEET proteins from cucumber (CsSWEET5a) and Arabidopsis (AtSWEET1 to AtSWEET17). A phylogenetic tree was constructed using the neighbor joining method and the p-distance model via MEGA 7.0 software. The scale bar represents the evolutionary distance of the number of amino acid differences per site. Bootstrapping was performed with 1000 replicates, and the values on the branches are shown as %. The amino acid sequences of the SWEET proteins used for the analysis are listed in Table S3.

A putative *SWEET* gene was cloned from the total RNA of male cucumber flowers and named *CsSWEET5a* (GenBank accession no. OR900513) based on its close phylogenetic relationship with *AtSWEET5* (also called *VEX1*) from Arabidopsis and earlier work [11,22]. The full-length cDNA of *CsSWEET5a* is 1139 bp in length, in which the 5′-untranslated region is 175 bp, the open reading frame (ORF) is 717 bp, encoding 238 amino acids, and the 3′-untranslated region is 247 bp (Table S1). The molecular weight of *CsSWEET5a* was 26.93 kDa, the isoelectric point was 9.18, and the grand average hydropathicity was 0.69 (Table S1). To determine the gene structure of *CsSWEET5a*, we analyzed the distributions of the exons and introns. The genomic sequence of *CsSWEET5a* is 1617 bp long and comprises six exons and five introns (Supplementary Figure S1A). The MOTIF Search and DeepTMHMM analysis revealed that the CsSWEET5a protein has seven TMs that are divided into two MtN3/saliva domains (Supplementary Figure S1B,C). In addition, the multiple sequence alignment revealed that the amino acid sequence of *CsSWEET5a* was highly homologous to that of *SWEET5* members of other plant species, all of which had seven TMs (Figure 1A and Table S2). Taken together, these results indicate that *CsSWEET5a* is a member of the *SWEET* gene family. To understand the evolutionary relationship between *CsSWEET5a* and the 17 *SWEETs* of Arabidopsis, a phylogenetic analysis was conducted. Based on the phylogenetic analysis, *CsSWEET5a* belongs to clade II of the *SWEET* gene family and has the closest relationship with *AtSWEET5* (60.58% amino acid sequence identity) (Figure 1B and Table S2).

### 2.2. CsSWEET5a mRNA Accumulates at High Levels in Developing Anthers and Pollen Cells

**Figure 2:**
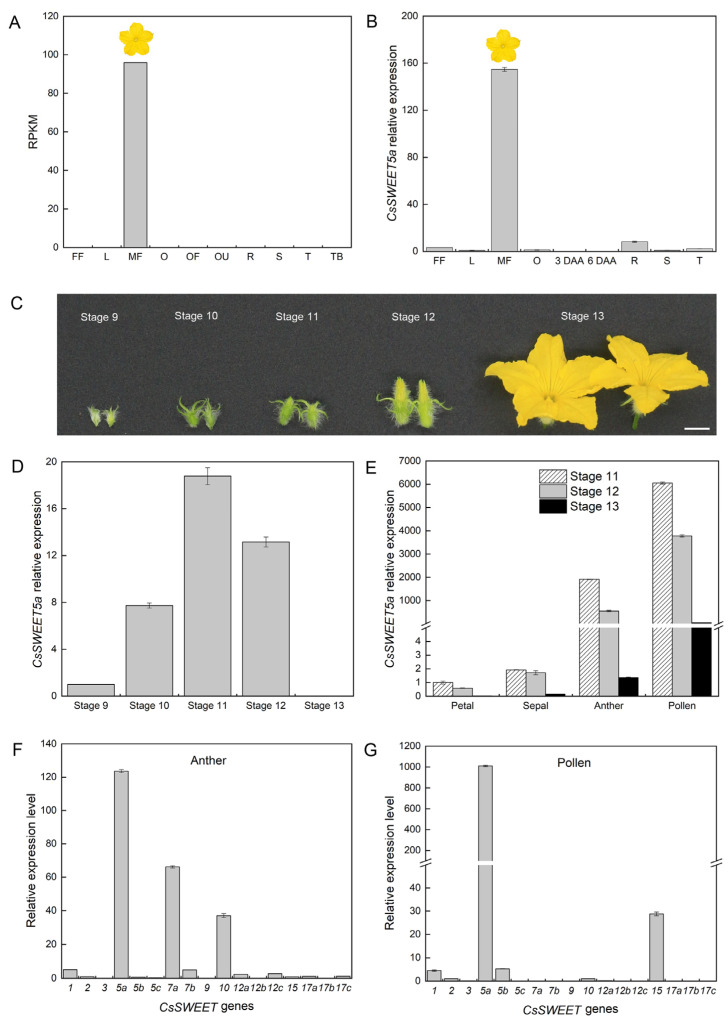
Expression of *CsSWEET* genes in cucumber organs. (**A**) Expression analysis of *CsSWEET5a* in different cucumber organs according to the transcriptome data. Raw data were obtained from the Cucurbit Expression Atlas (http://cucurbitgenomics.org/rnaseq/home (accessed on 2 December 2022)) under the project PRJNA80169. RPKM, reads per kilobase per million mapped reads. (**B**) Expression analysis of *CsSWEET5a* in different cucumber organs by quantitative real-time PCR (qRT–PCR). (**C**) Images of whole male flowers at various developmental stages. The stage division of male cucumber flowers was performed essentially as described previously [40], with slight modifications. The main features of each developmental stage were clarified in Table S4. Scale bars: 5 mm. (**D**) qRT–PCR analysis of *CsSWEET5a* in whole male flowers at various developmental stages. (**E**) Expression analysis of *CsSWEET5a* in various organs of male flowers at stages 11, 12, and 13. (**F**,**G**) Expression analysis of 17 *CsSWEET* genes in anthers (**F**) or pollen cells (**G**) that were isolated from male flowers at stage 11. A monoecious cucumber inbred line, C49, was used in (**B**–**G**). Error bars (**B**,**D**–**G**) represent the SEs from three biological replicates. FF, whole female flower at the time of opening; L, leaf; MF, whole male flower at the time of opening; O, ovary; OF, ovary_fertilized; OU, ovary_unfertilized; R, root; S, stem; T, tendril; TB, tendril base; 3 DAA, fruit at 3 days after anthesis; 6 DAA, fruit at 6 days after anthesis.

**Figure 3:**
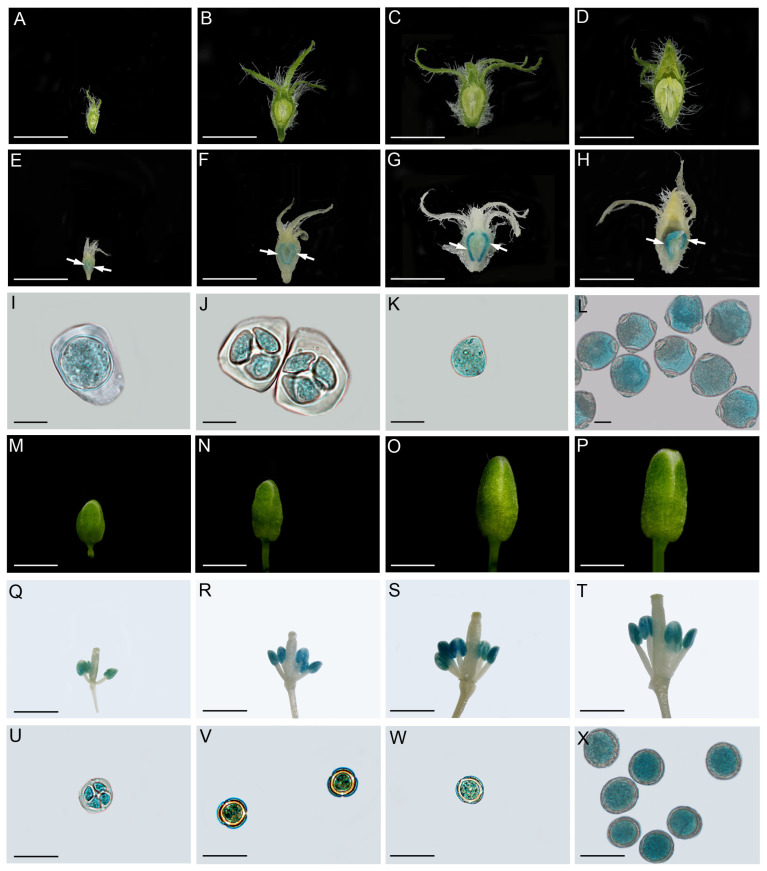
Histochemical staining of *β-glucuronidase* (*GUS*) activity in flower buds of cucumber and Arabidopsis plants. (**A**–**L**) Transient expression of *pCsSWEET5a::GUS* in male cucumber flowers from stages 9 to 12. Predominant *GUS* staining was observed in the anthers (indicated by arrows) at every stage of male flower development but not in the sepals or petals (**E**–**H**). Strong *GUS* activity was also observed in microsporocytes (**I**), tetrad microspores (**J**), uninuclear microspores (**K**), and mature pollen (**L**). (**A**–**H**) show the same male cucumber flower buds before and after *GUS* staining, respectively. (**A**,**E**) stage 9; (**B**,**F**) stage 10; (**C**,**G**), stage 11; (**D**,**H**) stage 12. (**M**–**X**) *GUS* staining of flower buds from T_2_ *pCsSWEET5a::GUS* transgenic Arabidopsis plants at different developmental stages. Dominant *GUS* expression was detected in anthers but not in sepals, petals, pistils, filaments, or peduncles (**Q**–**T**). To better visualize *GUS* expression in anthers, the sepals and petals in which *GUS* staining was not observed (Supplementary Figure S2) were removed from (**Q**–**T**). *GUS* staining was also strongly detected in the tetrad microspores (**U**), uninuclear microspores (**V**), bicellular pollen (**W**), and tricellular pollen (**X**) of the *pCsSWEET5a::GUS* transgenic Arabidopsis plants. (**M**–**T**) show the same Arabidopsis flower buds before and after *GUS* staining, respectively. Scale bars: 5 mm (**A**–**H**); 20 µM (**I**–**L**,**U**–**X**); 1 mm (**M**–**T**).

The RNA sequencing (RNA-Seq) data showed that *CsSWEET5a* was specifically expressed in whole male flowers at anthesis (Figure 2A), which was similar to our quantitative real-time PCR (qRT–PCR) results (Figure 2B). We further analyzed the expression pattern of *CsSWEET5a* in whole male flowers at different developmental stages via qRT–PCR (Figure 2C,D and Table S4). The *CsSWEET5a* expression increased rapidly during male flower development, peaked at stage 11, was maintained at a high level at stage 12 and then sharply decreased at stage 13 (at anthesis) (Figure 2D). At stages 11, 12, and 13, *CsSWEET5a* was predominantly expressed in anthers, especially in pollen cells, but was expressed at very low levels in the petals and sepals (including the receptacle and nectary) (Figure 2E). In addition, qRT–PCR was performed for all 17 *CsSWEET* genes using cDNA from anthers or pollen cells that were isolated from the male flowers at stage 11. The expression of *CsSWEET5a* in the cucumber anthers and pollen cells was markedly greater than that of all the other *CsSWEET* genes (Figure 2F,G). Next, *β-glucuronidase* (*GUS*) staining was performed. Strong GUS expression was observed in the anthers and microsporocytes/microspores/pollen grains of the male cucumber flowers from stage 9 to stage 12, which are the final four key stages at which the pollen matures in male cucumber flowers, namely, the microsporocyte stage (stage 9), meiosis stage (stage 10), uninuclear pollen stage (stage 11), and mature pollen stage (stage 12) (Table S4), but not in the petals and sepals (Figure 3A–L). Similar results were obtained for the unopened flower buds of T_2_ Arabidopsis plants transformed with the *pCsSWEET5a::GUS* construct (Figure 3M–X and Figure S2).

### 2.3. CsSWEET5a Is a Plasma Membrane Protein

**Figure 4:**
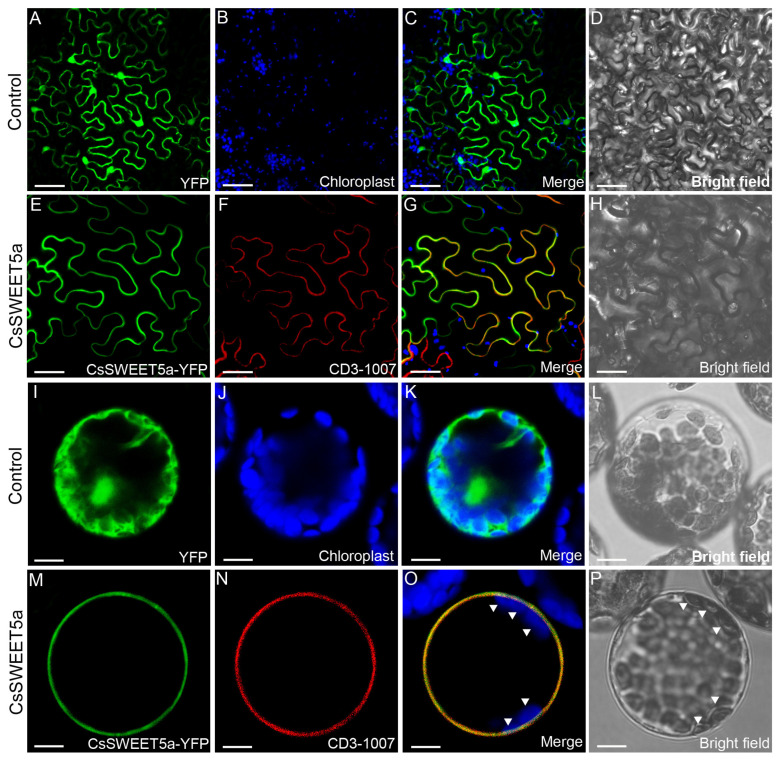
Subcellular location of the CsSWEET5a-YFP fusion protein in tobacco (*Nicotiana benthamiana*) leaf epidermal cells (**A**–**H**) and Arabidopsis mesophyll protoplasts (**I**–**P**). An empty vector expressing untargeted YFP was used as a control (**A**–**D**,**I**–**L**). The white arrowheads in (**O**,**P**) indicate chloroplasts. An mCherry-labeled marker (CD3-1007) was used to mark the plasma membrane position. The merged image shows YFP (green), chlorophyll (blue), and plasma membrane marker (red) fluorescence. The bright-field images are also presented. These images demonstrated that CsSWEET5a-YFP-derived fluorescence colocalized with the fluorescence of plasma membrane markers in a lining outside the chloroplast, indicating localization to the plasma membrane. Scale bars: 50 μm (**A**–**D**); 30 μm (**E**–**H**); 10 µM (**I**–**P**).

To assess the subcellular localization of CsSWEET5a, the CsSWEET5a-YFP fusion protein was transiently expressed in tobacco (*Nicotiana benthamiana*) leaf epidermal cells and Arabidopsis mesophyll protoplasts. As shown in Figure 4A–D,I–L, the epidermal cells of tobacco and mesophyll protoplasts of Arabidopsis plants transformed with the vector containing only YFP displayed fluorescence throughout their structures. When these epidermal cells and mesophyll protoplasts were coexpressed with CsSWEET5a-YFP and the mCherry-labeled plasma membrane marker (PM-rk; CD3-1007), yellow fluorescence resulting from the overlap of the green signals (derived from CsSWEET5a-YFP fusion proteins) and the red signals (derived from plasma membrane markers) was observed (Figure 4E–H,M–P). Additionally, we found that the green signals were located outside the chloroplasts, as indicated by the blue autofluorescence of the chloroplasts (arrowheads, Figure 4O,P). These results indicated that CsSWEET5a was localized to the plasma membrane.

### 2.4. CsSWEET5a Mediates Glucose and Fructose Transport in Yeast

**Figure 5:**
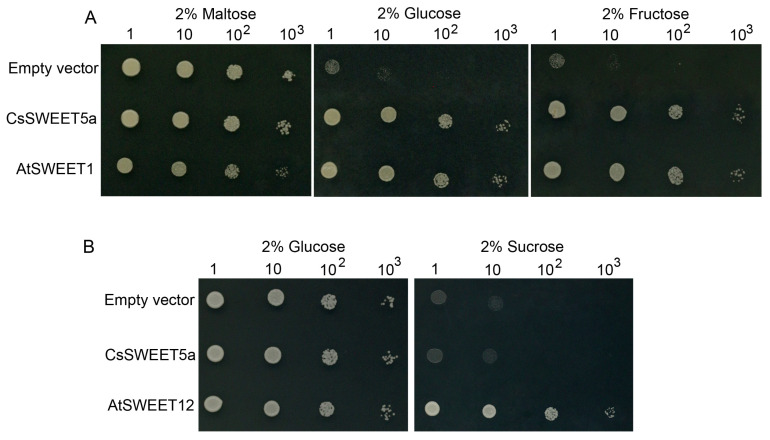
Analysis of the transport activity of *CsSWEET5a* in yeast. (**A**) Transport activity of *CsSWEET5a* in the yeast mutant EBY.VW4000. Yeast cells expressing an empty vector (negative control), *CsSWEET5a,* or *AtSWEET1* (positive control) were serially diluted (10-fold) and cultured on solid synthetic deficient media without uracil (SD-Ura) supplemented with 2% (*w*/*v*) maltose, 2% (*w*/*v*) glucose, or 2% (*w*/*v*) fructose as the sole carbon source. *CsSWEET5a* and *AtSWEET1* complemented the glucose and fructose uptake deficiency of EBY.VM4000, but the empty vector did not. (**B**) Transport activity of *CsSWEET5a* in the yeast mutant SUSY7/*ura3*. Yeast cells expressing an empty vector (negative control), *CsSWEET5a* or *AtSWEET12* (positive control) were serially diluted (10-fold) and cultured on solid SD-Ura media supplemented with 2% (*w*/*v*) glucose or 2% (*w*/*v*) sucrose as the sole carbon source. *AtSWEET12* complemented the sucrose uptake deficiency of SUSY7/*ura3*, but the empty vector and *CsSWEET5a* did not. Images were captured after incubation at 30 °C for 3–5 days.

*CsSWEET5a* belongs to the clade II *SWEET* gene family, the members of which generally mediate hexose transport [19], such as tomato *SlSWEET5b* and Arabidopsis *AtSWEET8* [6,11]. Thus, the transport function of *CsSWEET5a* was first investigated using the hexose uptake-deficient yeast (*Saccharomyces cerevisiae*) mutant EBY.VM4000, which cannot grow on hexose-containing media but can grow on maltose-containing media [41]. As expected, yeast cells expressing *CsSWEET5a* or *AtSWEET1* (used as a positive control [29,33]) grew well on media supplemented with glucose or fructose (Figure 5A). In contrast, yeast cells transformed with the empty vector (used as a negative control) grew very poorly on hexose-containing media (Figure 5A). This finding strongly indicated that *CsSWEET5a* encodes a hexose transporter.

Several studies have shown that *SWEET* genes outside clade III mediate sucrose transport [28,42]. Therefore, we further examined whether *CsSWEET5a* could transport sucrose by using the sucrose uptake-deficient yeast mutant SUSY7/*ura3*, which cannot grow on sucrose-containing media but can grow on glucose-containing media [43]. A drop test showed that yeast cells expressing *AtSWEET12* (used as a positive control [9]) grew well on media supplemented with sucrose (Figure 5B). However, neither the empty vector nor *CsSWEET5a* restored the growth of the yeast mutant SUSY7/*ura3* (Figure 5B). This suggests that *CsSWEET5a* cannot transport sucrose. Taken together, these results revealed that *CsSWEET5a* is a hexose transporter rather than a sucrose transporter.

### 2.5. Overexpression of CsSWEET5a Could Significantly Rescue the Fertility of atsweet8 Mutant Arabidopsis Plants

The *atsweet8* mutant Arabidopsis plants exhibit a severely reduced male fertility phenotype, as indicated by their markedly shorter siliques and dramatically reduced seed yield, which result from low pollen viability due to insufficient glucose supply [11,15]. Fortunately, *atsweet8* mutant Arabidopsis plants can propagate in a homozygous state because their fertility is partially recovered at the late reproductive stage [15]. To investigate the function of *CsSWEET5a*, we expressed the ORF of *CsSWEET5a* in *atsweet8* mutant Arabidopsis plants under the control of the cauliflower mosaic virus (CaMV) 35S promoter, and 17 independent *CsSWEET5a*/*atsweet8*-overexpressing (*CsSWEET5a*/*atsweet8*-OE) transgenic lines were obtained. Lines 3 and 6, which presented markedly greater *CsSWEET5a* transcript levels than did the other 15 lines, were selected to generate T_3_ homozygous transgenic lines (namely, OE-3 and OE-6) for further experiments (Supplementary Figure S3).

**Figure 6:**
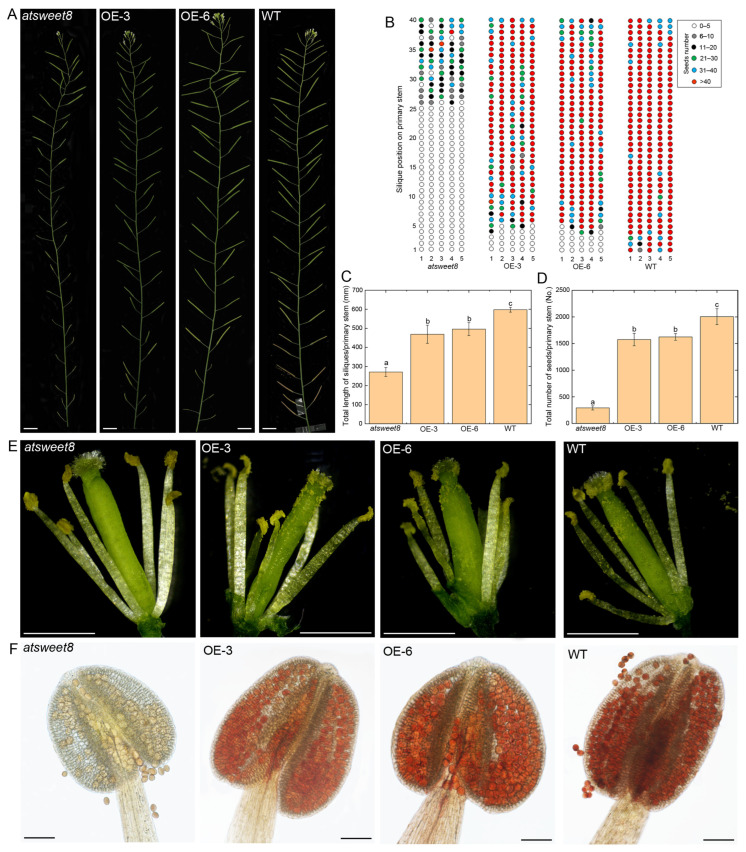
Characterization of *atsweet8* mutant Arabidopsis plants overexpressing *CsSWEET5a*. (**A**) Inflorescences of the primary stems of *atsweet8*, *CsSWEET5a*/*atsweet8*-overexpressing (*CsSWEET5a*/*atsweet8*-OE) transgenic lines, and the wild type (WT). (**B**) The position and corresponding seed number of siliques generated from the primary stem of the *atsweet8*, *CsSWEET5a*/*atsweet8*-OE, and WT plants. Five primary stems were randomly selected for presentation. (**C**,**D**) Total length of siliques (**C**) or total number of seeds (**D**) per primary stem of the *atsweet8*, *CsSWEET5a*/*atsweet8*-OE, and WT plants. The data are presented as the means ± SDs of ten primary stems. The different letters above the bars indicate significant differences (*p* < 0.01) determined by Duncan’s test. The siliques generated from the 1st to 40th flowers on the primary stem of the *atsweet8*, *CsSWEET5a*/*atsweet8*-OE, and WT plants were statistically analyzed, and the results are shown in (**B**–**D**). (**E**) Morphological features of the flowers of the *atsweet8*, *CsSWEET5a*/*atsweet8*-OE, and WT plants. Note that many more pollen grains were observed on the stigmas of the *CsSWEET5a*/*atsweet8*-OE lines than on those of the *atsweet8* mutant plants. (**F**) Triphenyl tetrazolium chloride (TTC) staining of anthers from the *atsweet8*, *CsSWEET5a*/*atsweet8*-OE, and WT lines. The red-stained pollen grains are viable and fertile. The flowers from the 10th to 20th on the primary stem were used to obtain the results shown in (**E**,**F**). Scale bars: 1 cm (**A**); 1 mm (**E**); 100 µm (**F**).

As shown in Figure 6, the *atsweet8* mutant and *CsSWEET5a*/*atsweet8*-OE Arabidopsis plants exhibited significantly different phenotypes. In *atsweet8* mutant Arabidopsis plants, approximately twenty-five siliques generated from the 1st to approximately 25th flowers on the primary stem were very short, producing only one or no seeds (Figure 6A,B). The length and number of seeds on the siliques generated after the 25th flowering on the primary stem of the *atsweet8* mutant Arabidopsis plants recovered somewhat, but they were much lower than those of the wild-type (WT) plants (Figure 6A,B). In contrast, only three to five siliques were sterile on the OE-3 and OE-6 plants, and these siliques were generated from the first to third or fifth flowers on the primary stem (Figure 6A,B). Moreover, most of the siliques of the OE-3 and OE-6 plants could produce more than 40 seeds, which was slightly lower than that of the WT plants (Figure 6A,B). Further analysis revealed that the total length of the siliques per primary stem of the *atsweet8* mutant Arabidopsis plants was 45.38% that of the WT plants, whereas in the OE-3 and OE-6 plants, the total length of the siliques per primary stem increased to 78.46% and 82.97% that of the WT plants, respectively (Figure 6C). In addition, the total number of seeds per primary stem of the *atsweet8* mutant Arabidopsis plants was only 14.56% that of the WT plants, whereas in the OE-3 and OE-6 plants, the total number of seeds per primary stem increased to 78.52% and 80.96% that of the WT plants, respectively (Figure 6D). These data suggested that the overexpression of *CsSWEET5a* can significantly rescue the fertility of *atsweet8* mutant Arabidopsis plants, as indicated by their dramatically longer siliques and markedly greater seed yield (Figure 6A–D).

Given that the flowers before the 25th on the primary stem of *atsweet8* mutant Arabidopsis plants always have few pollen grains, most of which are aborted, resulting in male sterility [15], we compared the quantity and viability of the pollen grains between the *atsweet8* mutant and *CsSWEET5a*/*atsweet8*-OE Arabidopsis plants using flowers from the 10th to 20th on the primary stem. As shown in Figure 6E, many more pollen grains were observed on the stigmas of the two OE lines than on those of the *atsweet8* mutant plants. Furthermore, we used triphenyl tetrazolium chloride (TTC) staining to examine the viability of the pollen grains, which distinguishes fertile pollen grains from aborted pollen grains [5,38]. We observed that almost all the pollen grains of the two OE lines, as well as those of the WT plants, were stained red, suggesting that they were viable and fertile (Figure 6F). In contrast, the pollen grains of the *atsweet8* mutant plants could not be strained by TTC solution (Figure 6F), which indicated that they were unviable and aborted. These data suggested that the overexpression of *CsSWEET5a* can significantly improve pollen viability and lead to the development of more fertile pollen grains, thereby rescuing the fertility of *atsweet8* mutant Arabidopsis plants.

## 3. Discussion

Pollen development, which is essential for plant sexual reproduction and crop yield, requires sugar support from anthers. The export of sugars from anther tissues into anther locules is a prerequisite for their uptake into developing pollen cells, but the molecular mechanisms by which sugars are released into anther locules are still poorly understood in all plant species except for Arabidopsis [15,16,17] and tomato [6]. In this study, we cloned the *CsSWEET5a* gene from male cucumber flowers and characterized its tissue-specific expression patterns, substrate specificities and cellular localization. Moreover, we generated *CsSWEET5a*-overexpressing transgenic Arabidopsis lines in the *atsweet8* mutant background and analyzed the contribution of *CsSWEET5a* to the export of hexoses from anther tissues into anther locules.

### 3.1. CsSWEET5a Encodes a Plasma Membrane Protein Highly Expressed in Developing Anthers and Pollen Cells

In the present study, we cloned a member of the *SWEET* gene family from cucumber, *CsSWEET5a*, and showed that it belongs to clade II and shares especially high homology with clade II members of other plant species (Figure 1 and Table S2). Notably, *AtSWEET8* in Arabidopsis [15] and *SlSWEET5b* in tomato [6], both of which belong to clade II, have been shown to play important roles in the export of hexoses from anther tissues into anther locules. Therefore, we further analyzed the spatial expression characteristics of *CsSWEET5a* using RNA-seq data, qRT–PCR, and *GUS* staining. *CsSWEET5a* was predominantly expressed in anthers and pollen cells from stage 9 to stage 12 (the final four key stages as the pollen matured in male cucumber flowers), while its expression was very low or undetectable in other tissues, such as sepals (including the receptacle and nectary), petals, female flowers, leaves, roots, stems, tendrils, and fruits (Figure 2 and Figure 3). Moreover, the transcript levels of *CsSWEET5a* in the anthers and pollen cells were markedly greater than those of all the other 16 *CsSWEET* genes found in the cucumber genome (Figure 2F,G). The high expression of *CsSWEET5a* in the cucumber anthers and pollen cells (Figure 2 and Figure 3) was similar to that of previously characterized sugar transporters that function in sugar supply during pollen development, such as tomato *SlSWEET5b* [6], Arabidopsis *AtSWEET8* [15], *AtSWEET13* [16,17], and cucumber *CsSUT1* [5]. As strong sinks, pollen cells’ sugar demands increase with development [1]. To fulfill the corresponding high sugar demand, sugars synthesized at increased levels in source leaves are transported into flowers through long-distance transport [1]. Consistent with this, Sun et al. [5] reported that the sugar content in male cucumber flowers increased from stage 9 to stage 11 and was maintained at a high level at stage 12. Notably, the developmental pattern of *CsSWEET5a* expression (Figure 2D) was similar to that of the sugar content in male cucumber flowers [5]. These positive correlations suggest that *CsSWEET5a* may be involved in sugar supply at the final four key stages (stages 9–12), as pollen matures in male cucumber flowers but not during subsequent pollen germination or pollen tube growth, which was indicated by the sharply decreased expression of *CsSWEET5a* at anthesis (stage 13; Figure 2D,E). Given that the structure of the anther wall, which consists of four layers (the epidermis, endothecium, middle layers, and tapetum), changes dynamically in a developmentally dependent manner [7,8], the exact cell layer in which *CsSWEET5a* is expressed across pollen development stages in male cucumber flowers needs further investigation.

Previous studies have confirmed that members of the *SWEET* gene family involved in apoplastic sugar exchange are usually located in the plasma membrane. For example, cucumber *CsSWEET7a*, involved in hexose apoplasmic unloading in fruits, receptacles, and nectaries [26,27]; *SlSWEET5b* [6], *AtSWEET8* [15], and *AtSWEET13* [16,17], involved in hexose or sucrose apoplasmic unloading from anther tissues to pollen cells; and *SlSWEET1a* [23], involved in glucose apoplasmic unloading in sink leaves, are located in the plasma membrane. In the present study, the colocalization of the CsSWEET5a-YFP fusion protein with a plasma membrane marker (CD3-1007) in tobacco leaf epidermal cells and Arabidopsis mesophyll protoplasts provided evidence that CsSWEET5a was located at the plasma membrane (Figure 4). Taken together, these findings show that the high expression of *CsSWEET5a* in the plasma membrane of the anthers and pollen cells supports its putative role in mediating sugar efflux across cell membranes between anther tissues and pollen cells during sugar apoplasmic unloading into developing pollen from stage 9 to stage 12.

### 3.2. CsSWEET5a Is a Glucose and Fructose Transporter That Complements the Function of AtSWEET8 in Arabidopsis

In general, members of the *SWEET* gene family that belong to clade II prefer to transport hexoses [19]. For example, *AtSWEET4*, which transports glucose and fructose, is expressed in the stele of the roots and veins of leaves and flowers and plays an important role in freezing and drought tolerance and in bacterial disease resistance [32]. *AtSWEET8* and *SlSWEET5b* have been shown to export glucose or glucose and fructose from anther tissues into anther locules to support pollen development, and plants in which these genes are mutated exhibit reduced male fertility [6,11,15]. In contrast to *AtSWEET4*, *AtSWEET8,* and *SlSWEET5b*, *OsSWEET5* has been found to transport galactose but not glucose or fructose [44]. The overexpression of *OsSWEET5* in rice alters the levels of sugar and auxin in leaves, resulting in growth retardation and premature senescence [44]. In the present study, the heterologous expression assay in the yeast EBY.VW4000 or SUSY7/*ura3* mutant showed that *CsSWEET5a* functions in the transport of glucose and fructose but not sucrose (Figure 5) or other hexoses, such as galactose [22].

Recently, Wang et al. [30] reported that *AtSWEET5* transports galactose and is highly expressed in pollen grains at flower stage 13 (at anthesis), but is not expressed in anthers. *AtSWEET5* plays a critical role in the early stages of pollen germination, and the pollen grains of the *atsweet5* mutant are tolerant to galactose inhibition during pollen germination in vitro [30]. In contrast to *AtSWEET5*, *CsSWEET5a* cannot transport galactose [22], and was predominantly expressed in anthers and pollen cells from stage 9 to stage 12, followed by a sharp decrease at stage 13 (Figure 2D,E). These differences in substrate specificity and tissue-specific expression pattern suggest that although *CsSWEET5a* is most closely related to *AtSWEET5* (Figure 1B), the biological function of *CsSWEET5a* in plants may differ from that of *AtSWEET5*. Notably, *AtSWEET8*, another member of the clade II *SWEET* gene family, exhibits characteristics similar to those of *CsSWEET5a* in terms of tissue-specific expression patterns, substrate specificities, and cellular localizations [11,15] (Figure 1, Figure 2, Figure 3, Figure 4 and Figure 5), which suggest that the biological function of *CsSWEET5a* may be similar to that of *AtSWEET8*. Therefore, we expressed the ORF of *CsSWEET5a* in *atsweet8* mutant Arabidopsis plants to investigate the function of *CsSWEET5a*. Compared with those of *atsweet8* mutant plants, the *CsSWEET5a*/*atsweet8*-OE transgenic plants produced many more pollen grains that were viable (Figure 6E,F), resulting in dramatically longer siliques and a much higher seed yield (Figure 6A–D). These results suggest that *CsSWEET5a* has sufficient ability to compensate for the loss of *AtSWEET8* function and thus significantly rescue the fertility of *atsweet8* mutant plants. In other words, similar to *AtSWEET8*, *CsSWEET5a* can export glucose from anther tissues and provide glucose for pollen development. In addition, since *CsSWEET5a* can also transport fructose, the possibility that *CsSWEET5a* plays a role in fructose efflux for pollen nutrition cannot be ruled out. In the present study, we carried out only ectopic expression of *CsSWEET5a* in *atsweet8* mutant Arabidopsis plants, and further studies on the overexpression or knockdown of *CsSWEET5a* in cucumber will help advance our understanding of the distinct role of *CsSWEET5a* in pollen development.

### 3.3. Hypothetical Model of Sugar Transport from Anther Tissues into Developing Pollen Cells in Cucumbers

The majority of sugars transported in the phloem of cucumber plants, a typical raffinose family oligosaccharide (RFO)-translocating species, are stachyose and raffinose [45]. However, the main sugars that accumulate in whole male cucumber flowers at stages 9–12 are sucrose and hexose (mainly glucose and fructose), with only small amounts of stachyose and raffinose detected [5]. Similar results have been observed for the receptacles and nectaries of male cucumber flowers at anthesis (stage 13) [26]. These studies suggest that stachyose and raffinose from source leaves may be metabolized into sucrose and hexose before they reach male flowers or quickly metabolized into sucrose and hexose after they reach male flowers. These possibilities are worthy of further investigation. Although the data focusing on the variations in the sugar content in cucumber anthers and pollen cells across pollen developmental stages are limited to date, one would assume that the dominant sugars in cucumber anther and pollen cells are sucrose and hexose, not stachyose or raffinose. Therefore, the unloading of sucrose and hexose from anther tissues of cucumber, a typical RFO-translocating species [45], is essential for pollen development, as is the case for sucrose-transporting plants.

**Figure 7:**
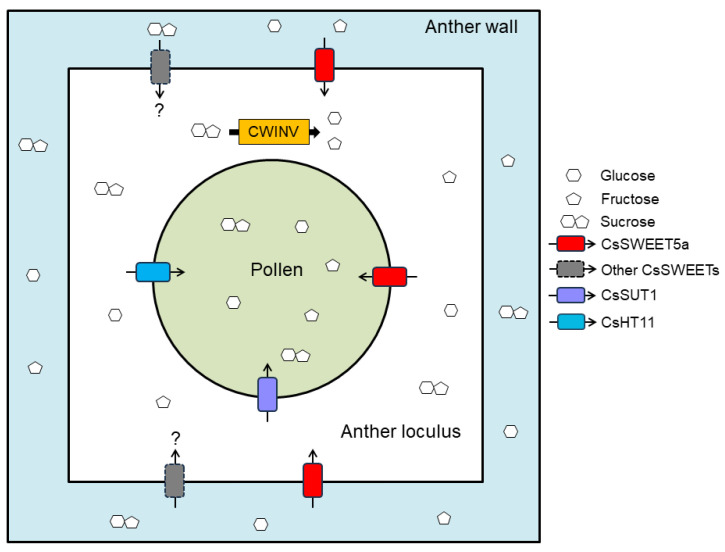
Hypothetical model of the role of *CsSWEET5a* in sugar transport from anther tissues into developing cucumber pollen at stages 9–12. CWINV, cell wall invertase.

Based on the above analysis and previous studies, we propose a model to illustrate the role of *CsSWEET5a* in transporting sugars from anther tissues into developing cucumber pollen cells at stages 9–12 (Figure 7). To fulfill the high sugar demand of developing pollen cells, large amounts of sugars are transported to male cucumber flowers. Once sugars are transported into anther tissues, cucumber *SWEETs* may export them into the anther locule (e.g., *CsSWEET5a* may export glucose and fructose, and other *CsSWEETs* which use sucrose as a substrate and localize to the plasma membrane of anther tissues may export sucrose). The released sucrose may be partially converted into glucose and fructose by cell wall invertase (CWINV) in anther locules [3,26,46]. Subsequently, glucose and fructose, together with sucrose that has not been hydrolyzed, may be infused from the anther locule into developing pollen cells by cucumber *SUTs*/*SUCs* (e.g., *CsSUT1* [5]), *SWEETs* (e.g., *CsSWEET5a*), or *MSTs* (e.g., *CsHT11* [47]) which are located in the plasma membrane of pollen cells. Although the characterization of *CsSWEET5a* provides insights into its role in supplying hexose to developing pollen cells via the apoplast, many aspects of this gene family remain poorly understood. For example, are there transcription factors or proteins that interact with *CsSWEET5a* on the plasma membrane? Which *CsSWEET* gene mediates the efflux of sucrose from anther tissues into anther locules? How do the sugar transporters involved in the apoplasmic unloading of sugars from anther tissues into developing cucumber pollen cells cooperate?

## 4. Materials and Methods

### 4.1. Plant Materials and Growth Conditions

A monoecious cucumber inbred line, C49, was used in this study. Seeds were sterilized, germinated, and grown in a greenhouse from March to July in Beijing, China. For qRT–PCR analysis, different organs (female flowers, leaves, ovaries, fruits, roots, stems, and tendrils) and different stages of male flowers (stages 9–13) were obtained from 3-month-old plants. The stage division of male cucumber flowers was performed essentially as described previously [40], with slight modifications. The main features of each developmental stage are clarified in Table S4. The separation of anther tissues and pollen cells from male cucumber flowers at stages 11 to 13 was carried out according to Aouali et al. [48], with slight modifications. Briefly, anthers were detached from male cucumber flowers, cut transversely into 1 mm slices, and placed in a centrifuge tube containing RNA-free water. After vortexing for 1 min, the mixture was filtered through nylon grids (200 µm mesh) to separate the fractions of anther tissues and pollen cells. The anther tissue fraction was rinsed with RNA-free water three times to remove any remaining pollen cells. The pollen cell fraction was centrifuged at 8000× *g* for 10 min, after which the supernatant was carefully pipetted off. All the samples were immediately frozen in liquid nitrogen and stored at −80 °C before analysis.

The Arabidopsis WT and *atsweet8* mutant plants used in this study were of the Columbia ecotype (Col-0). The Arabidopsis mutant *atsweet8*, a homozygous T-DNA insertion mutant (SALK_142803C), was obtained from the Arabidopsis Biological Resource Center. Surface-sterilized Arabidopsis seeds were sown on half-strength Murashige and Skoog (1/2 MS) solid media supplemented with 1% (*w*/*v*) sucrose and 0.25% (*w*/*v*) gellan gum (Phytotech, Lenexa, USA). After being kept at 4 °C for 3 days, the seeds were moved to a growth chamber (22 °C day and night temperature, 16 h light/8 h dark regime of 125 µmol quanta m^−2^ s^−1^ light intensity), in which they were grown for 7–10 days. The Arabidopsis plants were subsequently transplanted into a peat-based soil mixture (Pindstrup, Ryomgaard, Denmark) and grown in the growth chamber described above.

### 4.2. RNA Extraction, cDNA Synthesis, and qRT–PCR

RNA extraction, cDNA synthesis, and qRT–PCR were performed as described previously [33]. The primers used for qRT–PCR are shown in Table S5. Three biological replicates were included in the analysis. The relative transcript level of each target gene was calculated using the formula 2^−∆∆Ct^.

### 4.3. Cloning, Sequence Alignment, and Phylogenetic Analysis of CsSWEET5a

The full-length sequence of *CsSWEET5a* was amplified from the cDNA of male cucumber flowers at stage 12 using gene-specific primers (Table S5). The PCR products were inserted into the *p*EASY-T1 vector (TransGen, Beijing, China) and sequenced. The amino acid sequences of related SWEET proteins were obtained from the National Center for Biotechnology Information (http://www.ncbi.nlm.nih.gov (accessed on 5 January 2023)) or The Arabidopsis Information Resource (http://www.arabidopsis.org (accessed on 5 January 2023)) (Table S3). Multiple sequence alignment was performed via Clustal Omega (http://www.clustal.org (accessed on 10 March 2023)) and ESPript 3.0 (http://espript.ibcp.fr/ESPript/ESPript (accessed on 10 March 2023)). The phylogenetic tree was constructed using the neighbor joining method via MEGA 7.0 software, in which a bootstrap of 1000 replicates, p-distance, and pairwise deletion were applied.

### 4.4. Isolation of the Promoter Region of CsSWEET5a and GUS Expression Analysis

To generate the *pCsSWEET5a::GUS* construct, the *CsSWEET5a* promoter fragment (1998 bp) was amplified from the genomic DNA of male cucumber flowers at stage 12 using the primers listed in Table S5. The PCR products were subsequently inserted into the entry vector pDONR207 via a Gateway BP reaction and subsequently into the destination vector pMDC163, which carries the *GUS* gene, via a Gateway LR reaction, resulting in the *pCsSWEET5a::GUS* construct. The reconstructed plasmid was subsequently transformed into *Agrobacterium tumefaciens* strain GV3101. *A. tumefaciens*-mediated transformation of male cucumber flowers was performed as described previously [49], with slight modifications. Briefly, 1/2 MS liquid media containing *A. tumefaciens* cells harboring *pCsSWEET5a::GUS* was injected into the central parts of male cucumber flowers at different developmental stages using a 1 mL syringe. Cucumber plants were allowed to grow for 24-36 h under normal conditions, after which the injected male flowers were sampled for *GUS* staining. The *pCsSWEET5a::GUS* construct was also transformed into WT Arabidopsis plants using the floral dip method [50]. Samples of male cucumber flowers and transgenic Arabidopsis plants (T_2_ lines) were incubated in *GUS* staining solution (Huayueyang, Beijing, China) at 37 °C for 10 h or 3 h, respectively. After staining, the samples were dehydrated through an ethanol series and imaged with a digital camera (Canon EOS 70D, Tokyo, Japan), stereoscope (Olympus SZ61,Tokyo, Japan), or microscope (Zeiss Axio Imager Z2, Oberkochen, Germany).

### 4.5. Subcellular Localization of CsSWEET5a

To examine the subcellular localization of CsSWEET5a, the ORF of *CsSWEET5a* without a stop codon was amplified using specific primers (Table S5) and inserted into the expression vector pX-YFP_GW via Gateway technology. Arabidopsis protoplasts were subsequently transformed with the resulting CsSWEET2-YFP fusion construct as described previously [23]. The CsSWEET2-YFP fusion construct was further transformed into *A. tumefaciens* strain GV3101, which was subsequently infiltrated into the lower surface of tobacco leaves with a syringe, as previously described by Sugiyama et al. [42]. An mCherry-labeled plasma membrane marker (PM-rk; CD3-1007) was cotransformed with the CsSWEET2-YFP fusion construct in Arabidopsis protoplasts and tobacco leaves to determine the plasma membrane position. Fluorescence signals were examined via a confocal laser scanning microscope (Leica TCS SP8, Hesse, Germany).

### 4.6. Complementation Analysis of CsSWEET5a in Yeast

Yeast functional complementation assays of *CsSWEET5a* were also conducted as described previously [5,23,33]. Briefly, the ORF of *CsSWEET5a* was amplified via PCR and inserted into the yeast expression vector pDRf1-GW via the Gateway technique, yielding the pDRf1-GW-CsSWEET5a construct. Two positive control genes, *AtSWEET1* [29,33] and *AtSWEET12* [9], were also inserted into pDRf1-GW. The PCR primers used are listed in Table S5. Subsequently, the recombinant vectors and the pDRf1-GW empty vector (as a negative control) were separately transformed into the sucrose uptake-deficient yeast strain SUSY7/ura [43] or the hexose uptake-deficient yeast strain EBY.VW4000 [41] using the lithium acetate method. The transformants were incubated at 30 °C on synthetic deficient media without uracil (SD-Ura), supplemented with 2% (*w*/*v*) maltose/glucose/fructose/sucrose as the sole carbon source, and images were captured after 3–5 days of growth.

### 4.7. Ectopic Expression of CsSWEET5a in Arabidopsis

To overexpress *CsSWEET5a* under the control of the CaMV 35S promoter, the ORF of *CsSWEET5a* was amplified with gene-specific primers (Table S5). The resulting fragment was inserted into the binary vector pMDC32 via Gateway technology, as described by Guo et al. [51]. The resulting overexpression construct was subsequently transformed into *A. tumefaciens* strain GV3101, which was subsequently introduced into homozygous Arabidopsis *atsweet8* mutant plants as described previously [49]. The transgenic plants were screened on 1/2 MS solid media supplemented with 25 mg/L hygromycin.

### 4.8. Characterization of Plant Phenotype and Fertility

Inflorescences of the primary stems of eight-week-old plants were photographed with a Canon digital camera (EOS 70D). The length of the siliques was measured with Vernier calipers, and the number of seeds on the siliques along the primary stem was counted. For each line, ten plants were randomly selected, and 40 siliques on the primary stems of each plant were used for measuring silique length and counting seed number. Flower images were taken using a stereoscope (Olympus SZ61, Tokyo, Japan).

Pollen viability was determined by TTC staining, as previously described [38], with slight modifications. Briefly, anthers were detached from flowers before dehiscence and placed on a microscope slide containing 1% (*w*/*v*) TTC solution. After incubating at room temperature for 2 h, the anthers were observed and photographed under visible light using a microscope (Zeiss Axio Imager Z2, Oberkochen, Germany). For each line, ten plants were randomly selected, and 5 flowers from the 10th to 20th on the primary stem of each plant were randomly harvested for TTC staining. Viable pollen grains were stained red, while aborted pollen grains could not be stained by TTC solution.

### 4.9. Statistical Analyses

The data are presented as the means ± SEs of three or ten independent experimental replicates. Statistical analyses were performed using Excel 2016 software (Microsoft, Redmond, WA, USA). Significant differences were evaluated using Duncan’s test at the 1% level (*p* < 0.01) via SPSS 17.0 statistical software (IBM, New York, NY, USA). Histograms were generated using Origin 2018 software (OriginLab, Northampton, MA, USA).

## 5. Conclusions

In summary, *CsSWEET5a* is a plasma membrane hexose transporter highly expressed in the anthers and pollen cells of male cucumber flowers from the microsporocyte stage (stage 9) to the mature pollen stage (stage 12). In addition, *CsSWEET5a* can compensate for the loss of *AtSWEET8* function; that is, *CsSWEET5a* mediates the efflux of hexose from anther tissues and provides hexose for pollen development, thereby significantly rescuing the pollen viability and fertility of *atsweet8* mutant Arabidopsis plants.

## Data Availability

Data are contained within the article and supplementary materials.

## Supplementary Materials

The supporting information can be downloaded at https://www.mdpi.com/article/10.3390/ijms25021332/s1.
