# Supplementary material for: Functional Characterization of CsSWEET5a, a Cucumber Hexose Transporter That Mediates the Hexose Supply for Pollen Development and Rescues Male Fertility in Arabidopsis
Source: Int J Mol Sci. 2024 Jan 22;25(2):1332. doi: 10.3390/ijms25021332 (PMC10816302; doi:10.3390/ijms25021332)
Supplement: Supplementary file 1 [file ijms-25-01332-s001.zip › Figure S1.pdf]

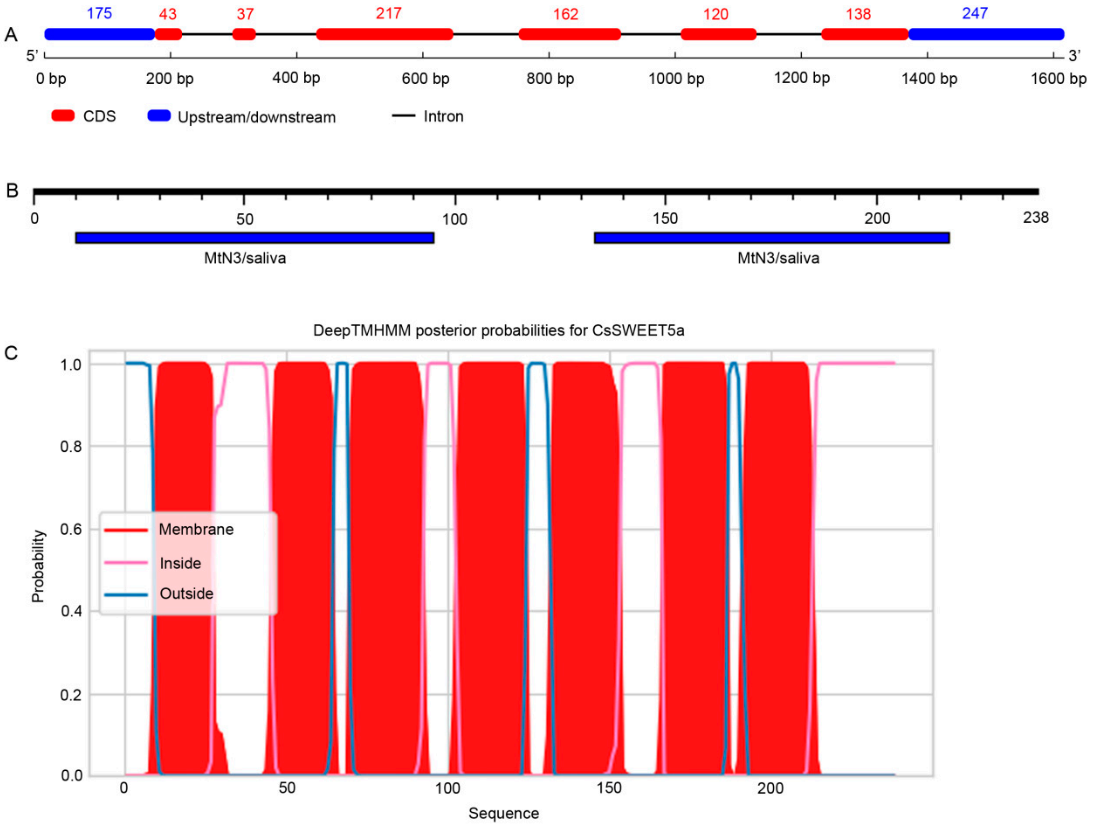

Figure S1. Structure, motif and transmembrane domain (TM) analysis of *CsSWEET5a*. (A) The exon–intron structure of *CsSWEET5a* was analyzed using the web-based bioinformatics tool Gene Structure Display Server (GSDS, http://gsds.gao-lab.org). Exons and introns are shown as red boxes and black lines, respectively. Upstream and downstream untranslated regions are shown as blue boxes. The numbers above the boxes indicate the nucleotide numbers of the upstream and downstream untranslated regions, as well as each exon. Scales are shown at the bottom of the diagram. (B) Conserved motifs of CsSWEET5a were analyzed by MOTIF Search (https://www.genome.jp/tools/motif). (C) TMs of the CsSWEET5a protein. The prediction website was DeepTMHMM (https://dtu.biolib.com/DeepTMHMM).
