# Supplementary material for: Functional Characterization of CsSWEET5a, a Cucumber Hexose Transporter That Mediates the Hexose Supply for Pollen Development and Rescues Male Fertility in Arabidopsis
Source: Int J Mol Sci. 2024 Jan 22;25(2):1332. doi: 10.3390/ijms25021332 (PMC10816302; doi:10.3390/ijms25021332)
Supplement: Supplementary file 1 [file ijms-25-01332-s001.zip › Figure S2.pdf]

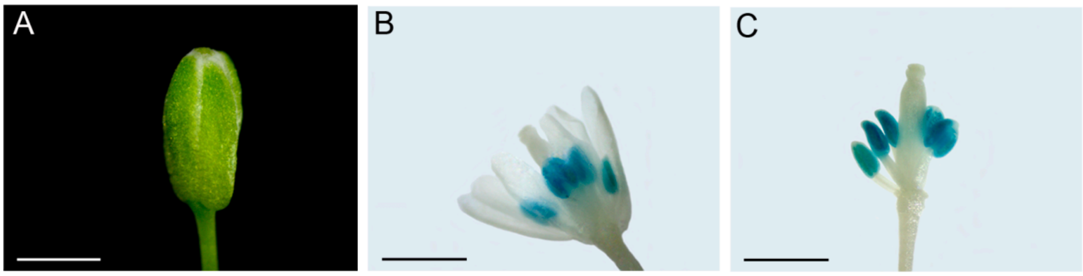

Figure S2. Histochemical staining of *β-glucuronidase* (*GUS*) activity in the flower buds of *pCsSWEET5a::GUS* transgenic Arabidopsis plants. Predominant *GUS* staining was observed in the anthers but not in sepals, petals, pistils, filaments, or peduncles. A and B are the same flower bud before and after *GUS* staining, respectively. C was obtained from B, whose sepals and petals were removed. Scale bars: 1 mm.
