# Supplementary material for: Functional Characterization of CsSWEET5a, a Cucumber Hexose Transporter That Mediates the Hexose Supply for Pollen Development and Rescues Male Fertility in Arabidopsis
Source: Int J Mol Sci. 2024 Jan 22;25(2):1332. doi: 10.3390/ijms25021332 (PMC10816302; doi:10.3390/ijms25021332)
Supplement: Supplementary file 1 [file ijms-25-01332-s001.zip › Figure S3.pdf]

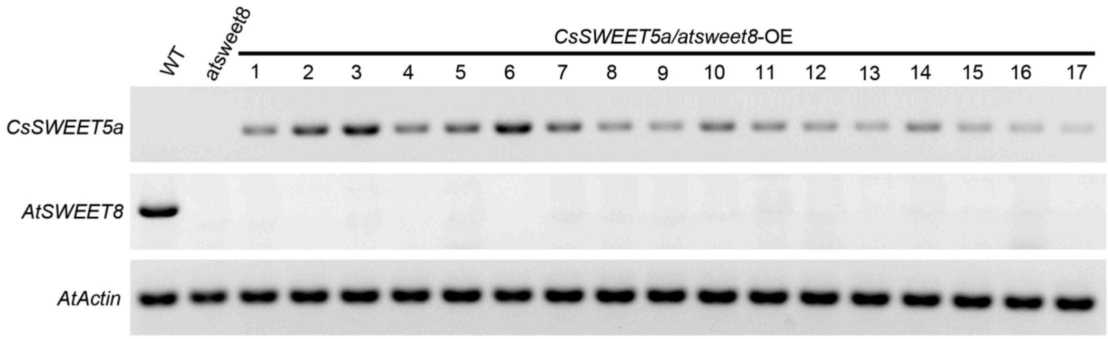


**Figure S3.** Reverse transcription PCR (RT-PCR) analysis of *CsSWEET5a* and *AtSWEET8* expression in the wild-type (WT), *atsweet8* mutant, and transgenic *CsSWEET5a/atsweet8*-overexpressing (*CsSWEET5a/atsweet8*-OE) lines. The *CsSWEET5a/atsweet8*-OE lines 3 and 6, which presented markedly greater *CsSWEET5a* transcript levels than did the other 15 lines and in which *AtSWEET8* expression was not detected, were selfed two times to generate $T_3$ homozygous transgenic lines.
